# Component features, odor‐active volatiles, and acute oral toxicity of novel white‐colored truffle *Tuber japonicum* native to Japan

**DOI:** 10.1002/fsn3.1325

**Published:** 2019-12-12

**Authors:** Tomoko Shimokawa, Akihiko Kinoshita, Norihisa Kusumoto, Shota Nakano, Noritaka Nakamura, Takashi Yamanaka

**Affiliations:** ^1^ Department of Forest Resource Chemistry Forestry and Forest Products Research Institute Tsukuba Japan; ^2^ Kyushu Research Center Forestry and Forest Products Research Institute Kumamoto Japan; ^3^ Department of Mushroom Science and Forest Microbiology Forestry and Forest Products Research Institute Tsukuba Japan

**Keywords:** chemical composition, *Tuber japonicum*, volatile compounds, White truffle

## Abstract

Component analysis of a novel white‐colored truffle native to Japan, *Tuber japonicum*, was performed to determine its characteristic features. The analysis of odor‐active volatile compound showed a high contribution of 1‐octen‐3‐ol and 3‐methyl‐2,4‐dithiapentane to the odor of *T. japonicum*. Although 2,4‐dithiapentane is a key odorant of well‐known white truffle *T. magnatum*, 3‐methyl‐2,4‐dithiapentane was detected from the ripe *T. japonicum*. The chemical components of *T. japonicum* showed no clear difference with those of edible truffles *T. magnatum* and *T. melanosporum*. It was rich in crude protein, crude fiber, and minerals (especially potassium), and low in crude fat. Glutamine and glutamic acid were detected in *T. japonicum* as free amino acids, while *T. magnatum* contained a large amount of alanine. Acute oral toxicity tests showed no abnormality, with an LD_50_ value of over 2000 mg/kg under the test conditions. The present study may support future market distribution of *T. japonicum* as a high‐class foodstuff.

## Graphical abstract

Component analysis of a novel white‐colored truffle native to Japan, *Tuber japonicum*, was performed to determine its characteristic features. The analysis of odor‐active volatile compound showed a high contribution of 1‐octen‐3‐ol and 3‐methyl‐2,4‐dithiapentane to the odor of *T. japonicum*. By confirming the safety and clarifying its characteristics of this new white‐colored truffle, it is expected to be a high‐class foodstuff in the future.

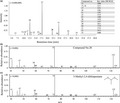

## 1 INTRODUCTION

Edible mushrooms are valuable sources of nutrition, including some mycorrhizal mushrooms that are not readily available on the market, but are considered premium seasonal food ingredients for local cuisine (Boa, 2004; Chang, 1980). Therefore, these edible mycorrhizal mushrooms are recognized as precious resources with a high global market value (Hall, Yun, & Amicucci, 2003; Wang & Hall, 2004). Truffles, the fruiting bodies of the genus *Tuber* belonging to Ascomycota, are widely known as high‐class food materials. Among them, Périgord black truffle (*Tuber melanosporum* Vittad.) and Italian white truffle (*Tuber magnatum* Pico) are well‐known premium truffles owing to their particular aromas and are traded globally. Although *T. melanosporum* is now cultivated in New Zealand and North America in addition to its native area (Europe), cultivation technique for *T. magnatum* has yet to succeed (Patel, 2012; Pierre, 2009; Zambonelli & Bonita, 2012).

In Japan, DNA barcoding and molecular phylogenetic analyses have confirmed the existence of more than 20 *Tuber* species (Kinoshita, Sasaki, & Nara, 2011). Among them, *Tuber japonicum,* Sasaki, A. Kinosh., & Nara, is a newly identified species with peculiar morphologically characteristics, including two‐spored asci and pale yellow irregularly reticulate ascospores. The novelty of this truffle has also been precisely confirmed by molecular analysis (Bonito et al., 2013; Kinoshita, Sasaki, & Nara, 2016).

As *T. japonicum* is little known in Japan, this white‐colored truffle has a limited culinary history, having been tasted by only a few people in a limited area. A large proportion of theoretically edible mushroom species have low consumption values, with many having an unverified edibility status (Kotowski, 2016; Sitta & Davoli, 2012). Although many species belonging to genus *Tuber* are considered edible, no information is available on *T. japonicum*. Japan has a tradition of picking edible wild mushrooms, with many mushrooms enthusiasts entering mountainous areas to collect wild mushrooms in the season. White‐colored truffle *T. japonicum* has attracted attention as a potential food material with commercial value along with increasing interest in distinctive local ingredients. To achieve future utilization as a food material, the characterization and safety confirmation of *T. japonicum* will be indispensable.

In this study, the chemical composition of Japanese white‐colored truffle *T. japonicum* was compared with those of two well‐known edible truffles, namely white truffle *T. magnatum* and black truffle *T. melanosporum*. To demonstrate its characteristic features, the free amino acid compositions and aroma compounds of Japanese white truffle collected from different locations were compared with those of *T. magnatum*. Furthermore, an acute oral toxicity test was conducted as the first step in safety confirmation for the future market distribution of *T. japonicum*.

## 2 MATERIALS AND METHODS

### 2.1 Samples

Fresh ascomata samples of *T. magnatum* from Italy and *T. melanosporum* from France were purchased in November 2017. Species of these market‐purchased samples was confirmed by analyzing the nucleotide sequences of the internal transcribed spacer (ITS) region of rDNA, as follows. Genomic DNA from each sample was extracted using a DNeasy Plant Mini Kit (Qiagen) according to the manufacturer protocol. The polymerase chain reaction (PCR) amplification was performed with the *T. melanosporum* ‐specific T.mel and T.mel_rev primer pair (Bonito, 2009), *T. magnatum* ‐specific P7 and M3 primer pair (Mello, Garnero, & Bonfante, 1999), and fungal universal ITS1F (Gardes & Bruns, 1993) and ITS4 (White, Bruns, Lee, & Taylor, 1990) primer pair as a positive control in a thermal cycler (Applied Biosystems 2720; Thermo Fisher Scientific) with slight modification. Ascomata samples of *T. japonicum* used in this study were as follows: Hyogo, collected in Hyogo Prefecture, Japan in November 2017; Tochigi 1, collected in Tochigi Prefecture, Japan in December 2017; Tochigi 2, collected in the same collection site of Tochigi 1 in November 2018; and Osaka, collected in Osaka Prefecture, Japan in December 2018. Ascomata samples of Tochigi 1 and Osaka were more matured than the other samples from their global color changes (Kinoshita et al., 2016). Ascomata samples from Hyogo, Tochigi 1, and Osaka were used for species identification based on the internal transcribed spacer (ITS) region. The PCR amplification was performed with the primer pair of ITS1F and ITS4 using a 2,720 thermal cycler after extraction of the genomic DNA. The DNA sequences were determined on ABI PRISM 3130xl Genetic Analyzer (Thermo Fisher Scientific). For species identification, the most similar sequences were searched with the nucleotide BLAST (http://www.ncbi.nlm.nih.gov/, GenBank, NCBI). Ascomata samples from Hyogo and Tochigi 1 showed 100% similarity with the data of LC410142 *T. japonicum* Ish1_Qa and LC388742 *T. japonicum* FFPRI 460518, respectively. Tochigi 1 sample showed 99.5% similarity with the data of LC410142 *T. japonicum* Ish2_Qa deposited in the GenBank database.

These raw ascomata samples were washed in running tap water using a soft brush to remove adhered soil and sand after sample arrival, and residual moisture was absorbed using dry paper. Worm‐eaten traces and badly damaged discolored parts were removed from the samples using a sterilized blade, and whole truffles were sliced. Individual specimens were used as samples for carbon, hydrogen, and nitrogen (CHN) analyses and moisture content analysis. Other samples were mixed to obtain a homogeneous sample. Samples used for proximate analyses, mineral analyses, and free amino acid component analyses were pulverized using a Millser IFM‐620DG blender (Iwatani, Tokyo, Japan) after lyophilization. Fresh samples used for volatile odor‐active compound analysis were sliced, quickly packed in glass bottles, frozen, and stored at −80°C until use. For acute oral toxicity tests, the fresh samples were homogenized in sterile endotoxin‐free water (Wako) using a homogenizer (with sterilized G10‐195ST generator probes inserted into an Omni TH‐01 homogenizer) to obtain specific concentrations of 0.5, 5, 30, and 200 mg/ml. These homogenates were stored at −80°C until use, and tests were carried out within one month of processing.

### 2.2 Chemical composition analysis

#### 2.2.1 Elemental CHN analysis

Elemental CHN composition data were obtained using a PerkinElmer 2,400 II CHN Elemental Analyzer.

#### 2.2.2 Proximate analysis

Sample moisture contents were measured by heating at 105°C and cooling in a desiccator to a constant weight. The ash content was measured at 600°C. Crude fat was extracted by Soxhlet extraction with diethyl ether, with reference to an AOAC Standard Method (1980, 7.056). Crude protein and crude fiber contents were measured using a facility service at the Japan Food Research Laboratory. The crude protein content was determined using the Kjeldahl method with a conversion factor of 6.25. Crude fiber was calculated using the ceramic fiber filter method based on the report of Sawaya, Al‐Shalhat, AlSogair, and Al‐Mohammad (1985). Crude protein, crude fat, crude fiber, and ash contents were each measured at least twice to obtain analytical results within the experimental error range of 5% and average values. Elemental CHN composition data were obtained using a PerkinElmer 2400 II CHN Elemental Analyzer.

#### 2.2.3 Mineral element analysis

Mineral measurements were conducted using the facility service at the Japan Food Research Laboratory. Mineral analysis of Na and K was conducted on samples digested with 1% hydrochloric acid by atomic absorption photometric analysis using a SpectrAA 240FS spectrometer (Agilent Technologies). For P, Fe, Ca, Mg, Zn, and Mn determination, the ash was hydrolyzed with 20% hydrochloric acid, and contents were analyzed by inductively coupled plasma (ICP) emission spectrometry using an Agilent 5100VDV spectrometer. Each mineral content was measured twice within the experimental error range of 5%, and an average value was obtained.

#### 2.2.4 Amino acid analysis

For free amino acid analysis, each powder sample (10.0 mg) was extracted with methanol/water (1.0 ml; 1:1, v/v) at room temperature for 2 hr with stirring. The precipitate obtained by centrifugation was washed again with fresh mixed solvent. The supernatants were combined and made up to 10 ml with methanol/water (1:1, v/v). A 2‐mL aliquot of this diluted solution was dried using an evaporator and resuspended with 500 µl of pH 2.2 lithium citrate buffer solution (Wako). After filtration through a 0.45‐µm membrane, all samples were analyzed using a JEOL JLC‐500/V2 amino acid analyzer. The following substances were used as standards: taurine, phosphoethanolamine, aspartic acid, threonine, serine, glutamic acid, glutamine, sarcosine, aminoadipic acid, glycine, alanine, citrulline, α‐amino butyric acid, valine, cysteine, methionine, cystathionine, isoleucine, leucine, tyrosine, β‐alanine, phenylalanine, β‐aminoisobutyric acid, γ‐aminobutyric acid, monoethanolamine, hydroxylysine, ornithine, histidine, lysine, 3‐methylhistidine, anserine, carnosine, arginine, asparagine, hydroxyproline, and proline.

#### 2.2.5 Odor‐active volatile analysis using GC–olfactometry (GC–O)/MS

Odor‐active volatile analysis was conducted using the facility service at the Hitachi Chemical Techno Service Co., Ltd. using a GERSTEL MPS‐2 autosampler (Gerstel) with a headspace and dynamic headspace system (DHS) option, a 7890 GC equipped with a 5977A Mass Selective Detector (Agilent Technologies), and a GERSTEL ODP3 olfactory detection port for GC–O. Each sample (0.14–0.18 g) was sealed in a 10‐ml vial bottle. For DHS analysis conditions, Tenax TA was used as an adsorbent at a trap temperature of 30°C. Each sample vial was incubated at 40°C for 5 min, purging with nitrogen (10 ml) at 10 ml/min, followed by a dry purge of nitrogen (100 ml, 50 ml/min) to reduce water content. Desorption of the compound was conducted using a thermal desorption unit (TDU) and programmable temperature vaporizing (PTV) injector inlet system. TDU analysis conditions were programmed to hold the temperature at 20°C for 0.5 min and then ramp to 250°C at a rate of 720°C/min, with a final time of 5 min. The PTV system was programmed to hold the temperature at 10°C for 0.5 min and then ramp to 250°C at a rate of 12°C/min, with a final time of 5 min. Compounds were separated using a capillary column (DB‐WAX, 60 m × 0.25 mm i.d., 0.25‐µm film thickness, J&W Scientific). The carrier gas was helium at a flow rate of 2.11 ml/min. The oven temperature was held at 40°C for 5 min and raised to 250°C at a rate of 12°C/min, with a final time of 5 min. The compounds were identified by matching their mass spectra with NIST 14 database software. Odor intensity was scored at six levels (from 0 to 5) by a trained panelist, as follows: 0, odorless; 1, perceptible odor; 2, weak smell with distinguishable odor; 3, easily perceptible odor; 4, strong odor; and 5, intense odor.

#### 2.2.6 Volatile compounds analysis by solid‐phase microextraction (SPME)‐GC/MS

Volatile compounds in truffle samples were extracted using a SPME fiber coated with 50/30 µm divinylbenzene/carboxen/polydimethylsiloxane (Supelco Co.). Frozen truffle sample was crushed by a mill, and headspace gas of 0.4 g of pulverized sample was extracted at 50°C for 20 min. The SPME fiber was then injected to the GC at 250°C in split mode (1:20 split ratio). The analysis of volatile compounds was carried out using a SHIMADZU QP‐2010 GC/MS Ultra System equipped with DB‐WAX UI capillary column (30 m × 0.25 mm i.d., 0.25‐µm film thickness, J&W Scientific). Helium was used as the carrier gas at a flow rate of 2.11 ml/min, and the column temperature was held at 40°C for 5 min and raised to 250°C at a rate of 5°C/min, with a final time of 5 min. The injector and detector temperatures were set at 250 and 230°C, respectively. The compounds were identified by matching their mass spectra with NIST 14 and FFNSC3 database libraries. Retention indices were calculated after analyzing C8‐C20 *n*‐alkane series (Merck) under the same chromatographic conditions.

#### 2.2.7 Acute oral toxicity

Acute oral toxicity tests against rats were conducted with reference to the OECD guideline for testing of chemicals 420 (17 December 2001: acute oral toxicity – fixed‐dose procedure) using the facility service at Public Interest Incorporated Foundation BioSafety Research Center (BSRC). The experiment involving the rat procedure was approved by the Animal Research Committee of Forestry and Forest Products Research Industry (FFPRI) and the BSRC in compliance with the ethical guidelines of both the FFPRI and BSRC.

Each test substance was prepared at the FFPRI and stored in a deep freezer immediately after preparation, as described previously. Test substances were sent to BSRC in a frozen state. Female Slc: Wistar [SPF] rats (7 weeks old; body weight range, 110–150 g) were purchased from Japan SLC Inc. and housed in a climate‐ and light‐controlled room with 12 hr each of light and dark. The test substance was administered to 8‐ to 9‐week old rats (body weight range, 138–148 g) that had been fasted overnight before administration. Starting doses of 300 mg/kg and 2000 mg/kg were selected for the sighting study owing to the lack of safety information and according to the guideline. A total of five animals were used in the main study. The test suspension was administered using a teflon gastric tube. For all animals, the general condition and body weight were observed for 14 days after administration, and all surviving animals were subjected to gross necropsy.

## 3 RESULTS AND DISCUSSION

### 3.1 Chemical composition

The proximate compositions of the three kinds of truffles are shown in Table 1. The protein contents of the *T. japonicum*, *T. magnatum,* and *T. melanosporum* samples analyzed in this study were comparable. Several studies have reported the chemical compositions of truffles, but few have been reported in English. The protein contents of white desert truffle *Tirmania nivea* (Desf.) (Trappe) from Saudi Arabia have been reported as 27.18% using the Kjeldahl method (N factor of 6.25) (Hussain & Al‐Ruqaie, 1999; Sawaya et al., 1985). The total soluble protein contents of *T. magnatum* and *T. melanosporum* have been reported as 24.0% and 8.7%, respectively (Saltarelli, Ceccaroli, Cesari, Barbieri, & Stocci, 2008), and the alkaline soluble protein content of *T. melanosporum* at maturation stage IV was 27.6% (Harki, Bouya, & Dargent, 2006). The validity of the protein contents of the three truffles in this study was also confirmed by elemental CHN analysis, as shown in Table 2. The protein contents of these three truffles, calculated with an N factor of 6.25, were 28.7% (*T. japonicum* Hyogo), 36.9% (*T. magnatum*), and 26.8% (*T. melanosporum*). The protein content of *T. japonicum* was between those of *T. magnatum* and *T. melanosporum*. The crude fiber of *T. japonicum* (14.4% and 12.2%) was also between those of *T. magnatum* (11.5%) and *T. melanosporum* (20.9%). The crude fiber contents of *T. japonicum* in this study were close to that reported for *Tir*. *nivea* (13.0%). The crude fat contents of the four truffle samples herein were in the range of 0.95%–1.34%, which were lower than the lipid content of *T. melanosporum* (7.8%) reported by Harki et al. (2006) using the method of Weete, Ssancholle, and Motant (1983). The crude fat contents of *Tir*. *nivea* from Saudi Arabia, as reported by Sawaya et al. (1985), ranged from 2.81% to 7.42%, despite using the same solvent, diethyl ether, as outlined in AOAC (1980) to compare the contents with that of *Tir*. *nivea*. The analytical results of fat contents in *T. latisporum*, *T. subglobosum*, and *T. pseudohimalayense* were 2.23%–2.55% with petroleum ether using a Soxhlet apparatus (Yan, Wang, Sang, & Fan, 2017). To confirm the crude fat contents of the samples used in this study, the three truffle samples were processed by Soxhlet extraction with petroleum ether. The resulting crude fat contents were 1.20% (*T. magnatum*), 1.11% (*T. melanosporum*), and 0.97% (*T. japonicum* Hyogo). As a result, the samples used in this study showed lower crude fat contents than those of truffles reported previously.

**Table 1:** Proximate composition of three kinds of truffles

	Moisture^a^	Crude protein^b^	Crude fiber^b^	Crude fat^b^	Ash^b^
*T. japonicum* (Hyogo)	78.8 ± 4.6	29.9	14.4	1.34	6.95
*T. japonicum* (Tochigi 2)	76.7 ± 2.2	31.1	12.2	1.12	8.32
*T. magnatum*	75.1 ± 1.8	38.5	11.5	1.13	9.41
*T. melanosporum*	73.3 ± 1.5	29.1	20.9	0.95	7.02

a Values are averages of three individual fresh specimens ± *SD* on a fresh weight basis (%).

b Values are averages of two determinations within the error range of 5%, in g/100 g dry weight basis.

**Table 2:** Elemental CHN composition of three kinds of truffles

	C (%)	H (%)	*N* (%)
*T. japonicum* (Hyogo)	42.04 ± 0.38	6.87 ± 0.07	4.59 ± 0.16
*T. japonicum* (Tochigi 1)	43.55 ± 1.28	6.95 ± 0.18	4.46 ± 0.58
*T. magnatum*	43.00 ± 0.72	7.04 ± 0.07	5.90 ± 0.58
*T. melanosporum*	43.28 ± 0.72	6.99 ± 0.09	4.29 ± 0.68

Note

Values are averages of three individual specimens ± *SD* on a dry weight basis.

Table 3 shows the minerals contents in the four truffle samples examined. Among these, the potassium content was markedly high, followed by the phosphorus content, as previously reported for other truffles (Harki et al., 2006; Sawaya et al., 1985). The potassium contents of *T. japonicum* were 683.9 and 650.1 mg/100 g dry weight, and those were lower than that of *T. magnatum* and *T. melanosporum* in this study. The potassium contents in this study were considerably lower than that of white truffle *Tir*. *nivea* (1734 mg/100 g dry weight, Sawaya et al., 1985). According to Harki et al. (2006), the potassium contents, expressed as % dry weight, of *T. melanosporum* ranged from 3.17% to 4.43%, measured using energy dispersive spectrometry. Libyan truffle *Terfezia boudieri* (Chatin) had a potassium content of 9,960 mg/kg on a moisture‐free basis (Ahmed, Mohamed, & Hami, 1981) by flame photometry. One reason for the lower potassium content in this study seemed to be the experimental conditions. Among other minerals, the sodium contents of *T. japonicum* were higher than that of *T. magnatum* and *T. melanosporum*, while its calcium contents were low.

**Table 3:** Mineral content of three kinds of truffles

	Na	P	Fe	Ca	K	Mg	Cu	Zn	Mn
*T. japonicum* (Hyogo)	45.0	171.0	1.93	3.54	683.9	14.4	1.71	7.82	0.07
*T. japonicum* (Tochigi 2)	61.8	223.0	1.04	4.10	650.1	16.8	1.58	5.16	0.06
*T. magnatum*	11.0	216.1	1.50	26.9	902.1	21.0	1.82	11.3	0.11
*T. melanosporum*	6.70	267.8	1.20	81.7	735.6	24.1	1.75	3.66	0.11

Note

Values are averages of two determinations within the error range of 5%, in mg/100 g dry weight basis.

The chemical composition of *T. japonicum* was found to be rich in crude protein, crude fiber, minerals, and low in crude fat, with no clear differences compared with the two controls. Although the nutritional value might vary with growth conditions, the characteristics of *T. japonicum* seemed not to deviate from those of the other two edible truffles of genus *Tuber* tested in this study.

### 3.2 Amino acid composition

Free amino acids detected in two samples of *T. japonicum* (Hyogo and Tochigi) and one sample of *T magnatum* are shown in Table 4. The amino acid components that exceeded contents of 10 µmol/g dry weight are listed. The most abundant amino acid detected from *T. japonicum* was glutamine, followed by glutamic acid. Alanine and asparagine were also detected in the extract of *T. japonicum*. In contrast, a significantly larger amount of alanine, over six times higher than that in *T. japonicum*, was detected from *T. magnatum*. For *T. melanosporum* at maturation stage V, glutamine was reported to be the most abundant free amino acid after cysteine, while alanine was the most abundant at ripening stage IV (Harki et al., 2006). Alanine and glutamine are known to be related to sweetness, while glutamic acid is the source of umami taste (Bachmanov et al., 2016; Schiffman & Sennewald, 1981). This combination of free amino acids might affect the characteristic flavor of each truffle.

**Table 4:** Free amino acids of three truffle samples

	*T. japonicum* (Hyogo)	*T. japonicum* (Tochigi 1)	*T. magnatum*
Aspartic acid	25.4 ± 1.04	11.43 ± 0.65	12.0 ± 0.46
Threonine	12.8 ± 0.38	6.56 ± 0.09	9.27 ± 0.20
Serine	16.7 ± 0.58	9.82 ± 0.42	14.97 ± 0.56
Asparagine	34.1 ± 2.12	10.6 ± 0.31	5.92 ± 1.37
Glutamic acid	68.5 ± 1.77	38.68 ± 1.31	50.4 ± 0.65
Glutamine	90.3 ± 2.23	49.34 ± 0.75	61.1 ± 1.16
Glycine	11.3 ± 0.32	4.93 ± 0.12	21.0 ± 0.32
Alanine	41.6 ± 1.31	23.9 ± 0.50	256 ± 3.13
α‐Amino butyric acid	1.73 ± 0.29	1.80 ± 0.13	22.8 ± 0.35
Valine	5.43 ± 0.18	11.3 ± 0.31	10.5 ± 0.07
Cystathionine	35.26 ± 0.99	26.4 ± 4.47	20.5 ± 0.65
Isoleucine	3.36 ± 0.12	12.1 ± 0.22	8.29 ± 0.87
Lysine	14.8 ± 0.90	1.77 ± 0.03	13.45 ± 0.62
Arginine	10.03 ± 2.48	0.34 ± 0.30	73.45 ± 3.21

Note

Values are averages of three determinations ± *SD* in µmol/g dry weight basis.

### 3.3 Volatile compounds

**Figure 1:**
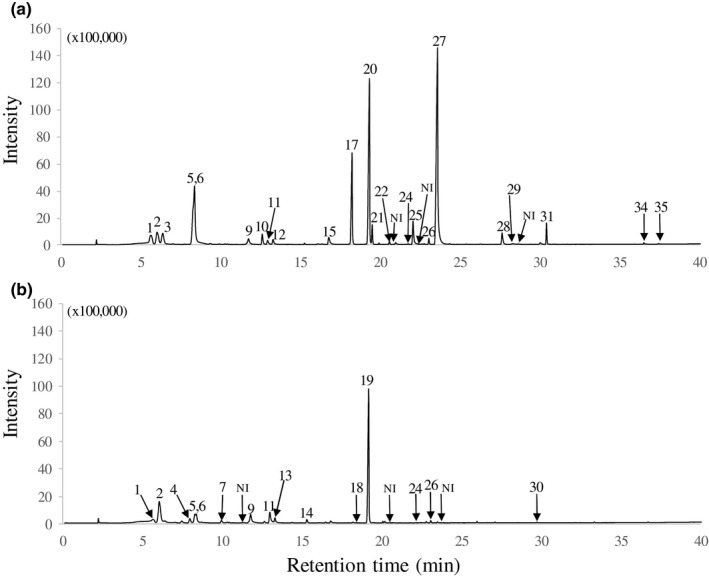
Total ion chromatograms of volatile compounds in *Tuber japonicum* Tochigi 1 (a) and *T. magnatum* (b) by GC–olfactometry/MS analysis. Compound numbers are listed in Table 5

Based on a library search identification of their mass spectra, 14, 26, and 19 compounds were detected in *T. japonicum* Hyogo, Tochigi 1, and Osaka samples by GC–olfactometry/MS analyses, respectively (Table 5). In the three *T. japonicum* samples, 1‐octen‐3‐ol was strongly detected. This component was also marked as level 5 in the sensory test, having the most intense perceptible odor. In addition to 1‐octen‐3‐ol, 3‐methyl‐2,4‐dithiapentane (1,1‐bis(methylthio)ethane), a sulfur volatile component, was also detected in the Tochigi 1 sample at an odor intensity of level 5 (Table 5). 3‐Methyl‐2,4‐dithiapentane was also detected at a level 3 odor intensity from the Osaka sample but not detected from the Hyogo sample. The ascomata samples from Tochigi 1 and Osaka were collected in December, while the Hyogo sample was collected in November. Delaying the collection time might cause advanced ripening and change the composition of volatile components. Other sulfur compounds, such as dimethyl sulfide, dimethyl disulfide, and dimethyl trisulfide, were also detected from the Tochigi 1 and Osaka samples. These sulfur compounds are found in many truffles and are considered to be characteristic aroma components especially in black truffles (Rubini, Belfiori, Riccioni, & Paolocci, 2012; Spivallo, Ottonello, Mello, & Karlovsky, 2011). The most characteristic detected odor of white truffle *T. magnatum* was 2,4‐dithiapentane, which had a level 5 odor intensity in this study as shown in Table 5. 2,4‐Dithiapentane is a sulfur volatile component well known as the key odorant of *T. magnatum* (Bellesia, Pinetti, Bianchi, & Tirillini, 1996; Fiecchi, Kienle, Scala, & Cabella, 1967). Instead of 2,4‐dithiapentane, 3‐methyl‐2,4‐dithiapentane was identified as a distinctive sulfur volatile component from *T. japonicum* (Figure 1). Therefore, the odor characteristics of *T. magnatum* and *T. japonicum* had obvious differences. To our knowledge, this is the first report of 3‐methyl‐2,4‐dithiapentane as the main component of truffle aroma.

**Table 5:** Volatile compounds identified in four truffle samples by GC–olfactometry/MS with accompanying sensory data

Compound No.	Compound	Odor intensity				Odor description
		*T. japonicum* (Hyogo)	*T. japonicum* (Tochigi 1)	*T. japonicum* (Osaka)	*T. magnatum*	
1	Dimethyl sulfide	–	3	1	3	rotten cabbage‐like
2	Propanal	–	3	–	3	pungent, fruity, roasted
3	2‐Methylpropanal	0	1	–	–	pungent
4	2‐Butanone	–	–	–	0	–
5	2‐Methylbutanal^a^	(3)^a^	(3)^a^	(3)^a^	(3)^a^	fruity, roasted
6	3‐Methylbutanal^a^					–
7	Pentanal	–	–	–	1	pungent, fruity, roasted
8	Diacetyl^b^	–	–	2	–	fatty, butter‐like
	NI	3	–	–	3	fishy
9	1‐Propanol	–	0	–	0	–
10	Dimethyl disulfide	–	1	1	–	rotten cabbage‐like
11	Hexanal	4	2	1	4	green, leafy
12	2‐Methyl−1‐propanol	–	0	–	–	–
13	2‐Methyl−2‐butenal	–	–	–	0	–
14	2‐Methyl−2‐pentanal	–	–	–	0	–
15	2‐Methyl−1‐butanol	–	0	–	–	–
16	3‐Methyl−1‐butanol	0	–	1	–	fermentation odor
17	3‐Octanone	0	1	0	–	mild fruity, herb‐like
18	Methyl propyl sulfide^b^	–	–	–	1	
19	2,4‐Dithiapentane	–	–	–	5	garlic‐like
20	3‐Methyl−2,4‐dithiapentane	–	5	3	–	rotten seaweed‐like
21	1‐Octen−3‐on^b^	3	3	4	–	mushroom‐like
22	Anisole	–	0	–	–	–
	NI	2	1	1	2	green, leafy
23	1‐Hexanol	0	–	–	–	–
24	Dimethyl trisulfide	–	3	3	3	rotten seaweed‐like
25	3‐Octanol	0	0	0	–	–
	NI	–	2	–	–	fragrant
26	2‐Octenal	3	3	3	3	fatty, nut‐like
27	1‐Octen−3‐ol	5	5	5	–	mushroom‐like
	NI	–	–	–	3	fragrant
28	2‐Octen−1‐ol^b^	–	0	0	–	–
29	Phenylacetaldehyde	–	2	–	–	honey‐like
	NI	–	2	–	–	rotten seaweed‐like
30	2,4‐Nonadienal	–	–	–	2	fatty
31	1,4‐Dimethoxybenzene	–	2	–	–	cream‐like
32	2,4‐Decadienal	1	–	2	–	fatty
33	Phenylethyl alcohol	–	–	2	–	rosy
34	Anisaldehyde	–	2	–	–	sweet
35	1,2,4‐Trimethoxybenzene	–	2	–	–	cream‐like

Abbreviations: –, not detected; NI, compound not identified.

a Individual discrimination was not possible because of overlapping peaks.

b Although strongly suggested, the estimated component.

**Figure 2:**
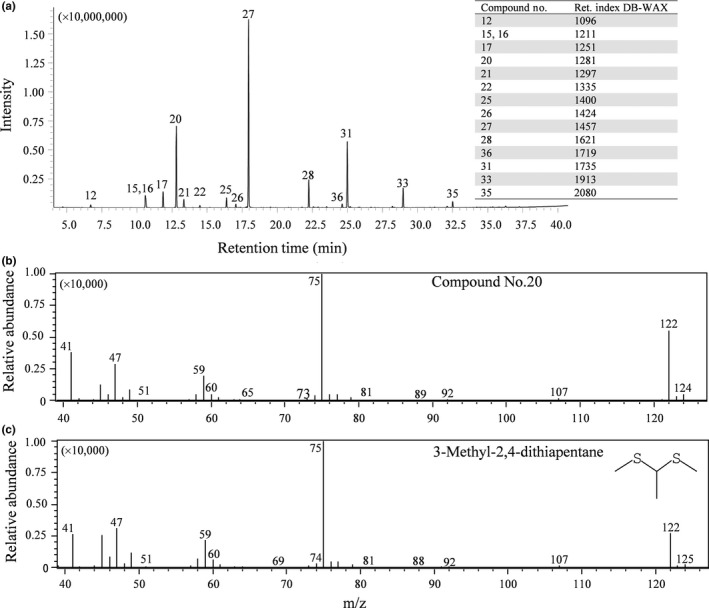
Analysis of volatile compound number 20 in *Tuber japonicum* Tochigi 1. (a): Total ion chromatogram of volatile compounds detected by SPME‐GC/MS analysis with retention index of DB‐WAX at the right space; (b): mass spectra identification of product 20; (c) 3‐methyl‐2,4‐ dithiapentane in NIST 14 library. Compound numbers are listed in Table 5

For further confirmation of 3‐methyl‐2,4‐dithiapentane (Compound No. 20) in *T. japonicum*, we conducted SPME‐GC/MS. The total ion chromatogram of *T. japonicum* Tochigi 1 is shown in Figure 2 along with retention indices. Among the detected 15 compounds by SPME‐GC/MS analysis, 3‐(methylthio)‐1‐propanol (Compound No. 36) was not detected in the GC–olfactometry/MS analysis. The most abundant peak was identified as 1‐octen‐3‐ol (Compound 27) similar to the result of Figure 1. Mass spectra of the second abundant peak, Compound 20, were compared to that of library data, and it was identified as 3‐methyl‐2,4‐dithiapentane within 95% similarity by NIST 14 library (Figure 2). We also analyzed the volatile compounds of *T. magnatum* at the same system and detected 2,4‐dithiapentane (Ret. Index 1279, DB‐WAX UI, data not shown).

Many reports have shown that truffle aroma varies depending on the ripening stage (Saltarelli et al., 2008; Zeppa et al., 2004), with some assessing the microorganisms involved in forming the truffle aroma (Splivallo et al., 2015; Vahdatzadeh, Deveau, & Splivallo, 2015). Since the aroma quality affects the market value of truffles, it is necessary to further characterize the changes in odor‐active components in *T. japonicum*.

### 3.4 Acute oral toxicity

The acute oral toxicity of *T. japonicum* was investigated using a fixed‐dose procedure. As truffles are a food ingredient that can be eaten raw, the raw truffle was pulverized in sterilized water using a homogenizer and frozen until administered. In the sighting study, no abnormalities were observed in individuals with doses of both 300 mg/kg and 2000 mg/kg, that let four additional test animals at 2000 mg/kg for the main study. Table 6 shows the weight shift of the tested animals. No death occurred during the observation period of 14 days, and no abnormalities were found in the general condition, weight shift, and autopsy report of the tested animals. Based on the above results, the LD_50_ value of *T. japonicum* at an acute oral toxicity against rat was determined to be over 2000 mg/kg under these test conditions.

**Table 6:** Body weights of rats treated once orally with *Tuber japonicum*

Test substance	Dose (mg/kg)	Animal ID No.	Days after administration		
			0^b^	7	14
*T. japonicum* (Hyogo)	300	F4^a^	138	167	183
	2,000	F2^a^	143	173	178
		F3	147	180	186
		F5	145	178	181
		F6	148	181	188
		F9	145	174	182
	Mean ± *SD*		146 ± 2	177 ± 4	183 ± 4

a Sighting study.

b Before administration.

## 4 CONCLUSION

Distinctive characteristics of Japanese white‐colored truffle *T. japonicum* were identified by odor volatile compounds analysis and free amino acid analysis, while chemical composition analysis showed similar features to those of well‐known edible *Tuber* species (*T. magnatum* and *T. melanosporum*). The generality of the chemical composition of *T. japonicum* is an important consequence of the overall characteristics as a member of genus *Tuber*. Acute oral toxicity tests showed no abnormalities, with an LD_50_ value of over 2000 mg/kg. This result provides partial safety information for *T. japonicum*, accompanied by the generality of its chemical composition. Regional foodstuffs need generality as an ingredient and distinguishable qualities from others. White‐colored truffle *T. japonicum* seems to satisfy these demands and has a potentially high market value that may drive future market distribution.

## CONFLICT OF INTEREST

The authors declare that they have no conflict of interest.

## ETHICAL APPROVAL

This study involving the rat procedure was approved by the Animal Research Committee of Forestry and Forest Products Research Industry (FFPRI) and the BSRC in compliance with the ethical guidelines of both the FFPRI and BSRC.
